# Comparison of clinical, capnography and ultrasonography methods for ETT placement: An observational study

**DOI:** 10.6026/973206300211808

**Published:** 2025-07-31

**Authors:** Rashmi Pal, Kamlesh Choudhary, Rashpal S. Gill

**Affiliations:** 1 Department of Anaesthesia, MGM Medical College and MY Hospital, Indore, Madhya Pradesh, India

**Keywords:** Endotracheal intubation, ultrasonography, capnography, auscultation, endotracheal tube (ETT) confirmation

## Abstract

Accurate confirmation of endotracheal tube (ETT) placement is essential to prevent airway complications. While capnography is the established standard, ultrasonography (USG) offers a rapid, non-invasive alternative. Hence, prospective study of 105 patients was completed. USG and capnography demonstrated excellent diagnostic accuracy, while auscultation showed slightly lower specificity. Therefore it is of interest to report ultrasonography as a faster and effective option for ETT confirmation in critical settings.

## Background:

Placement of Endotracheal tube (ETT) is a fundamental intervention in airway management during general anesthesia and critical care, crucial for maintaining oxygenation and ventilation [1]. However undetected esophageal intubation continues to pose a serious threat, occurring in approximately 6% of emergency intubations and 1.75% of elective procedures [2, 3]. Such errors can result in life-threatening outcomes, including hypoxemia, aspiration, cardiovascular collapse and death, particularly in vulnerable patient populations [4, 5, 6- 7]. To minimize such risks, confirmatory testing following intubation is strongly emphasized by the Advanced Cardiac Life Support (ACLS) guidelines [4]. Traditionally, clinicians rely on physical signs such as chest rise, tube fogging and auscultation of breath sounds [6]. Five-point auscultation and observation of bilateral chest movement are commonly used bedside techniques [8]. Yet, these clinical methods are subjective and their reliability may diminish in high-noise environments or during resuscitation [1, 2]. Capnography, which detects end-tidal carbon dioxide or ETCO_2_, is regarded as the established standard for confirming ETT placement due to its high diagnostic precision and real-time monitoring [9, 10-11]. Nonetheless, it has notable limitations in low perfusion states such as cardiac arrest, hypotension and pulmonary embolism, where false readings can occur [7, 12]. Furthermore capnography demands active ventilation and specialized equipment, which may not always be available in prehospital or resource-limited settings [13, 14]. USG has emerged as a viable alternative; it is portable, non-invasive and not dependent on pulmonary blood flow [2]. Techniques like visualizing tracheal rings, identifying the "double tract" sign and detecting bilateral lung sliding have shown promise in accurately distinguishing tracheal from esophageal intubation [7, 8]. Studies such as Lahiri *et al.* reported USG confirmation of ETT placement within 11.44 seconds compared to 21.68 seconds for capnography, with a sensitivity of 96.92% and specificity of 100% [1]. However despite its advantages, ultrasonography is still underutilized due to operator dependency and inconsistent training [2, 3-4]. Comparative validation in various clinical contexts remains necessary to establish its role alongside established methods. Therefore it is of interest to report a prospective observational study comparing the diagnostic accuracy and efficiency of the clinical method, capnography and ultrasonography for confirmation of correct ETT placement.

## Materials and Methods:

This prospective observational study was conducted in the Department of Anaesthesiology at MGM Medical College & M.Y. Hospital, Indore, over a 12-month period from October 1, 2023, to September 30, 2024. Ethical clearance was obtained from the Institutional Ethics Committee and written informed consent was secured from all participants in their vernacular language. A total of 105 adult patients between 20-60 years classified as ASA I or II and scheduled for elective surgery under general anesthesia requiring endotracheal intubation were included. Patients underwent preoperative assessment as per institutional protocol. Sample size was calculated using G*Power software (v3.1.9.2), assuming ultrasonography sensitivity of 97.8%, with a 95% confidence level and 3% margin of error. Accounting for 10% attrition the final sample size was set at 105.

## Inclusion criteria:

[1] Age 20-60 years

[2] ASA I or II

[3] Mallampati grade I / II

[4] Either sex

[5] Elective surgery requiring endotracheal intubation

## Exclusion criteria:

[1] Patient refusal

[2] Thyromental distance <6 cm

[3] Known or anticipated difficult airway

[4] Neck masses, facial trauma, cervical spine disease

[5] BMI ≥30 kg/m^2^

## Procedure:

Patients underwent standard pre-anesthetic evaluation, including airway assessment and routine investigations. All patients were kept nil per oral for six hours before surgery. Standard monitoring was applied in the operating room. Anesthesia was induced with glycopyrrolate (10 µg/kg IV), midazolam (0.02 mg/kg IV), fentanyl (2 µg/kg IV), propofol (1.5-2.5 mg/kg IV) and succinylcholine (2 mg/kg IV). ETT placement was performed via direct laryngoscopy by an experienced anesthesiologist.

Immediately after intubation, three blinded anaesthesiologists independently confirmed ETT placement using:

[1] Ultrasonography (USG): High-frequency linear probe (13-6 MHz, ALPIION) at the suprasternal notch. Tracheal intubation showed two hyperechoic lines, esophageal intubation presented the "double tract sign."

[2] Auscultation: Observation of bilateral chest rise and five-point auscultation at infraclavicular, infra-axillary and epigastric points.

[3] Capnography: Defined as a sustained square waveform with end-tidal CO_2_ >4 mmHg after five breaths using side stream capnograph. Capnography served as the established standard.

Confirmation time taken by each method was recorded. For esophageal intubation the tube was immediately repositioned, but only the first attempt was included for analysis.

## Outcomes:

Primary outcomes included diagnostic accuracy and time required for ETT confirmation. Secondary outcomes were sensitivity, specificity, PPV and NPV of each method against capnography.

## Statistical analysis:

Data were analyzed using SPSS v25.0. Continuous variables were expressed as mean ± SD; categorical variables as frequencies and percentages. Normality was tested using the Kolmogorov-Smirnov and Shapiro-Wilk tests. One-way ANOVA with Tukey's post-hoc test was used for group comparisons. Diagnostic performance was analyzed using 2x2 contingency tables. A p-value <0.05 was considered statistically significant.

## Results:

Out of 120 screened patients, 105 met the criteria and were enrolled for analysis. The study population had a male predominance (61%) and the majority were aged between 19-30 years (Table 1 - see PDF). All three methods auscultation, ultrasonography (USG) and capnography showed high efficacy in detecting correct ETT placement. Positive detection rates were 92.4% for auscultation and 91.4% for both USG and capnography (Table 2 - see PDF). Diagnostic performance analysis revealed that both USG and capnography achieved 100% sensitivity, specificity, positive predictive value (PPV) and negative predictive value (NPV). Auscultation showed 100% sensitivity and NPV, but slightly lower specificity (88.9%) due to one false-positive case (Table 3 - see PDF and Table 4 - see PDF). In terms of speed, USG confirmed ETT placement significantly faster (mean time: 8.98 minutes) than capnography (11.64 minutes) and auscultation (12.94 minutes), with statistically significant differences (p < 0.001) across all comparisons (Table 5 - see PDF).

## Discussion:

Accurate confirmation of endotracheal tube (ETT) placement is crucial to prevent hypoxia, aspiration and airway injury during airway management [1, 2]. Various techniques are available, including auscultation, capnography and ultrasonography (USG), each with distinct advantages and limitations [3, 4-5]. Identifying the most accurate and time-efficient modality remains a clinical priority [6]. In our study both USG and capnography demonstrated perfect diagnostic accuracy (100% sensitivity, specificity, PPV and NPV), whereas auscultation showed slightly lower specificity (88.9%) due to one false-positive case. These results align with Roy *et al.* who reported 100% sensitivity and specificity for both USG and capnography [7]. Similar findings were documented by Mekewar *et al.* who confirmed perfect accuracy with both USG and capnography, even when assessing tracheal, endo bronchial and esophageal intubations [15]. Comparable high diagnostic performance of USG has been validated in multiple studies. Kuppusamy *et al.* demonstrated that ultrasonography is faster and more accurate than capnography for ETT confirmation in critically ill patients with 98.36% sensitivity and 100% specificity [16]. Chowdhury *et al.* reported USG sensitivity of 99.17% and specificity of 100% [17]. Thomas *et al.* documented sensitivity and specificity of 97.89% and 100%, respectively, while Das *et al.* in a meta-analysis confirmed pooled sensitivity and specificity of 98% [18, 19]. Reddy *et al.* similarly observed 100% sensitivity and specificity using USG [20]. Indian settings is Abhishek *et al.* found USG sensitivity of 96.84% and specificity of 100%, strongly correlating with ETCO_2_ [2]. Adi *et al.* reported 98% sensitivity and 100% specificity for tracheal ultrasonography [21]. However the slightly lower accuracy was reported by Sim *et al.* (88.7%) likely influenced by emergency cases [22]. USG also proved superior in confirmation time. In our study, USG had a mean confirmation time of 8.98 minutes, significantly faster than capnography (11.64 min) and auscultation (12.94 min) (p < 0.001). This advantage is clinically important in emergencies, where delayed confirmation may risk hypoxia and complications. Mekewar *et al.* observed faster USG confirmation (16.87 ± 8 sec) compared to auscultation and capnography (24.7 ± 10.6 sec) [15]. Roy *et al.* also reported faster USG confirmation (4.9 ± 1.09 sec) [7]. Thomas *et al.* and Sethi *et al.* further supported USG's time efficiency [8, 17].

Interestingly the authors Abhishek *et al.* found capnography marginally faster (8.99 ± 1.04 sec) than USG (12.0 ± 1.32 sec) possibly due to their dual-scan protocol, while our study used a single transverse view [2]. Unlike capnography USG performance remains unaffected by pulmonary perfusion deficits, such as in cardiac arrest, hypotension or pulmonary embolism where capnography may yield false-negative results [5, 6]. USG's portability, real-time imaging and rapid learning curve make it a valuable tool for point-of-care use in perioperative and emergency settings [3, 4 and 12]. Demographic homogeneity in our study (predominantly ASA I-II patients aged 19-40 years) ensured minimal confounding [7, 8]. Similar demographic distributions have been reported in earlier studies, allowing better comparability of results [7, 8, 9- 10]. While our findings reinforce USG's role as a reliable, rapid alternative to capnography, certain limitations remain. The study was single-centered with a relatively small sample size, potentially limiting generalizability. Procedural variability and observer skill differences especially with auscultation may have influenced outcomes [15, 16]. Despite these our findings remain consistent with previously published literature. Our study supports USG as a highly accurate and time-efficient method for ETT confirmation. It holds particular promise for routine practice, resource-limited setups and emergency airway management.

## Conclusion:

Ultrasonography demonstrated diagnostic accuracy equivalent to capnography and was faster in confirming ETT placement. Its non-invasive, real-time application makes it especially useful in emergencies and settings with limited resources. USG is a practical and efficient tool that should be considered for routine airway confirmation.
